# Contribution of Massive Mitochondrial Fusion and Subsequent Fission in the Plant Life Cycle to the Integrity of the Mitochondrion and Its Genome

**DOI:** 10.3390/ijms22115429

**Published:** 2021-05-21

**Authors:** Ray J. Rose

**Affiliations:** School of Environmental and Life Sciences, The University of Newcastle, Callaghan, NSW 2308, Australia; ray.rose@newcastle.edu.au

**Keywords:** plant mitochondria, plant mitochondrial fusion, plant mitochondrial fission, plant mitochondrial DNA, massive mitochondrial fusion in plants, plant life cycle, mitophagy

## Abstract

Plant mitochondria have large genomes to house a small number of key genes. Most mitochondria do not contain a whole genome. Despite these latter characteristics, the mitochondrial genome is faithfully maternally inherited. To maintain the mitochondrial genes—so important for energy production—the fusion and fission of mitochondria are critical. Fission in plants is better understood than fusion, with the dynamin-related proteins (DRP 3A and 3B) driving the constriction of the mitochondrion. How the endoplasmic reticulum and the cytoskeleton are linked to the fission process is not yet fully understood. The fusion mechanism is less well understood, as obvious orthologues are not present. However, there is a recently described gene, *MIRO2*, that appears to have a significant role, as does the ER and cytoskeleton. Massive mitochondrial fusion (MMF or hyperfusion) plays a significant role in plants. MMF occurs at critical times of the life cycle, prior to flowering, in the enlarging zygote and at germination, mixing the cells’ mitochondrial population—the so-called “discontinuous whole”. MMF in particular aids genome repair, the conservation of critical genes and possibly gives an energy boost to important stages of the life cycle. MMF is also important in plant regeneration, an important component of plant biotechnology.

## 1. Introduction

**Figure 1:**
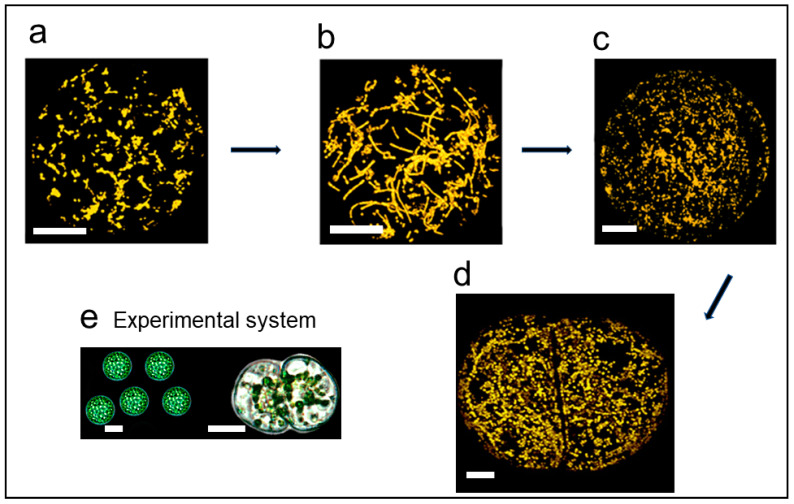
Mitochondrial fusion and fission prior to the first cell division in regenerating *Nicotiana tabacum* protoplasts. Visualised by GFP-expressing mitochondria. (**a**) Mitochondria in freshly isolated protoplasts are small ovoid organelles, showing some clumping. (**b**) Massive mitochondrial fusion forming highly elongated mitochondria. (**c**) After fusion, there is fission, generating large numbers of small mitochondria. (**d**) Uniformly dispersed mitochondria enable unbiased inheritance at cell division. (**e**) The experimental system. Dividing protoplast shows clustering of chloroplasts around the nucleus. Bars (**a**–**d**) 10 μm, Bars (**e**) = 20 μm. Figure 1 taken from [1] the Yale Journal of Biology and Medicine under Creative Commons Attribution-NonCommercial-ShareAlike 3.0.

Mitochondria in flowering plants, which this review focuses on, are the sites of oxidative phosphorylation, producing most of the cellular adenosine triphosphate (ATP), central to providing the energy for plant life processes [1]. In addition, mitochondria are the sites of a complex metabolism and synthesise important compounds, including vitamins [2,3,4]. Plant mitochondria are mostly observed as small spherical ovoid organelles (Figure 1), 0.2–2.0 µm in diameter [5,6]. In the major plant model, *Arabidopsis*, a 0.8-µm sphere is considered a reasonable representation of an average mitochondrion, when estimating protein copy numbers per mitochondrion [6]. In a typical mesophyll cell, there can be 300–600 of these average mitochondria [7,8]. Mitochondria are, however, pleomorphic and dynamic organelles that have other morphologies that are important in understanding a number of aspects of mitochondrial cell biology and molecular genetics as they undergo fission and fusion [9]. Fusion can lead to long tubular mitochondria of 16 µm [7] or large tubuloreticular mitochondria [10] in certain cells and under certain conditions.

Mitochondria contain DNA, which in *Arabidopsis* codes for 32 proteins, including critical proteins of the cytochrome electron transport chain, plus 3 rRNA and 22 tRNA genes [11,12]. However, nuclear DNA codes for the overwhelming majority of mitochondrial proteins. Isolated mitochondria from *Arabidopsis* cell suspension cultures contained 917 different protein species, when all contaminating proteins from other compartments were identified and eliminated [6]. All Angiosperms contain similar number of mitochondrial DNA (mtDNA) genes, but there is extensive variation in genome size. Most mitochondrial genomes in flowering plants range from 200 to 700 kb, but some can be up to 11 Mb [13], with most of the DNA being non-coding. This contrasts with mammalian mitochondria which range from 15 to 17 kb [13], with human mtDNA being 16.6 kb. Mitochondria do not form de novo and divide by fission to produce daughter mitochondria [14,15]. As mitochondria are semi-autonomous, with molecular evidence suggesting they were derived by endosymbiosis from an α-bacterium 1.5 billion years ago [16], it would be expected that there would be a regular transmission of DNA to daughter mitochondria. However, in a population of mitochondria, some contain less than a genome or even no DNA [8,9,17]. This problem can be overcome by fusion and subsequent fission [9,18]. Fission and fusion have been reviewed in Logan (2006) [19] and Arimura (2018) [20]. Fusion may involve mitochondrial pairs or may be massive, involving many mitochondria [21].

This review focuses on the current understanding of the cell biology and genes involved in mitochondrial fission and fusion, the cell and molecular biology of fission and fusion in the metabolizing cell, the cell cycle, development, and inheritance. How might these processes maintain the integrity/quality of the plant mitochondrion and its complex genome?

## 2. Mitochondrial Division

### 2.1. Proteins of the Mitochondrial Fission Machinery 

The first protein associated with fission by higher plant mitochondria was the dynamin-like protein ADL2b [14], identified by similarities to yeast DNM1pand human DRP1. The fluorescently labelled ADL2b (GFP-ADL2b) localises to the mitochondrial constriction sites and the tips of the mitochondrion. Subsequently, similar data were obtained for ADL2a, with GFP-ADL2a localised to constriction sites [22,23]. Mutants of ADL2a produce long tubular mitochondria [22,23], due to impairment of mitochondrial division. There are a number of dynamin-related proteins, which are large GTPases with diverse cellular functions [24]. A unified nomenclature for plant dynamin-related proteins has ADL2a and ADL2b named as DRP3A and DRP3B, respectively [24], which has become common usage [20,25]. DRP3A and DRP3B are also involved in peroxisome division [26].

Members of the DRP1 family have also been implicated in mitochondrial division [27], denoted here as DRP1C and E. Elongated mitochondria were produced in mutant plants with a T-DNA insertion in the DRP1E locus [27]. However, the function of the DRP1 family is in membrane trafficking for cell plate formation in cytokinesis and for cellular tip growth in pollen tubes and root hairs [28]. Mutants of the dynamin-related protein DRP5 also cause elongated mitochondria [26], though its role has now been shown to be in the division of the chloroplast [29] and peroxisome [30]. It is generally accepted that the dynamin-related proteins involved in driving mitochondrial fission are DRP3A and DPR3B [20,31,32]. Double mutants of DRP3A and DRP3B can produce extreme tubular mitochondria longer than 100 µm [31]. DRP3A and DRP3B are the contractile proteins that act as “molecular scissors”, forming a ring-like structure that divides the mitochondria into two [32].

In addition to DRP3A and DRP3B, there are several other proteins involved in the mitochondrial division machinery. Mutants of human and yeast orthologues of *FISSION* *1* and *FISSION 2* (*FIS1* and *FIS2*) enabled *BIGYIN1* and *BIGYIN2* (*FIS1* and *FIS2*) to be identified with a mitochondrial division role [33]. A role for *FIS1* and *FIS2* in mitochondrial division is supported by mutant and overexpression studies [34]. FIS1 proteins recruit and anchor DRP3A and DRP3B proteins to the outer mitochondrial membrane [32,34,35]. *ELONGATED MITOCHONDRIA1 (ELM1)* was identified in *Arabidopsis* by screening and analysing mutants with longer and fewer mitochondria [36]. The phenotype was similar to *drp3a*. Studies with ELM1:GFP showed fluorescence surrounding the mitochondria [36], rather than the constriction site and mitochondria tips found in DRP3A and DRP3B GFP studies. ELM1 also interacts with DRP3A and DRP3B. A plausible model [36] based on *Arabidopsis* data and understanding from yeast and humans is that ELM1 can interact with DRP3A or DRP3B and then interacts with FIS1 or FIS2, which anchors the complex to the mitochondrion. Deficiency of the mitochondrial phospholipid cardiolipin destabilises DRP3 proteins [37], which would affect the DRP3:ELM1:FIS complex, inhibiting fission [37]. Other proteins identified in plant mitochondrial fission are PEROXISOMAL AND MITOCHONDRIAL DIVISION FACTOR1 (PMD1) and PMD2 [38]. Mutants produced elongated and fewer mitochondria, and the PMD1 and PMD2 coiled coil proteins tethered to the outer mitochondrial membrane. However, there was no evidence of interaction with DRP3A/B or FIS1/2 [38]. At this stage, there is no specific role for PMD1 or PMD2 in the fission process. In addition, they have non-redundant roles [38]. One possibility is that these proteins are more strongly associated with morphogenesis than proliferation.

### 2.2. The Endoplasmic Reticulum and Mitochondrial Fission

It was first shown in yeast and mammalian cells that mitochondrial division occurred at sites where the mitochondria contacted endoplasmic reticulum (ER) tubules [39]. Some constriction occurs at the ER sites prior to recruitment of the dynamin-related protein (DRP1in mammals DNM1 in yeast) which forms a helical ring around the mitochondrion as DRP3A/3B would do [39]. Models of mitochondrial division in mammals and yeast have an ER tubule wrapping around the constriction site adjacent to the dynamin-related protein ring [40,41]. What do we know of the ER connection in plants? In *Arabidopsis*, using simultaneous visualisation of the ER and mitochondria, elongated mitochondria induced by physiological treatments and mutants were investigated [5]. Beads on string-like mitochondrial structures in association with ER tubules and small polygons were observed, such that this was the forerunner of the mitochondrial division process into smaller mitochondria ER tubules that could encircle the mitochondria. Studies on the moss, *Physcomitrella patens*, also found an association between the ER tubules and mitochondrial fission [42]. These observations suggest that ER–mitochondria interactions lead to assembly of the DRP3A/3B fission complex. There clearly is a dynamic relationship between the ER and mitochondria that influences mitochondrial division. The ER tubules in plants form and then shrink, and can be formed from cisternae and form polygons [43]. However, the specific role of the ER in plant mitochondrial division requires further work. Some clues will no doubt be found from comparative research carried out on mitochondrial dynamics with fungi and animals as well as bryophytes, as highlighted in the reviews by Arimura [20] and Logan [19].

In mammalian cells, actin is active at ER mitochondria contact sites [41]. Actin polymerisation is driven by the ER-associated inverted formin INF2 in the early stages of mitochondrial fission [44], with DRP1 completing fission and severing. In plant cells, the actin inhibitor Latrunculin B inhibited the dispersion of mitochondria and reduced mitochondrial number in dividing protoplasts [7]. Mitochondrial numbers were, however, hard to determine in the presence of Latrunculin B due to lack of dispersion and may have been underestimated. Oryzalin, the microtubule de-polymerising agent, did not inhibit mitochondrial dispersion and inhibited mitochondrial division, with mitochondria being larger. In *N. tabacum* BY-2 cells, microtubules are involved in mitochondrial fission in mitosis [45]. How the cytoskeleton in plants contributes to mitochondrial fission is still unclear. The situation in mammals has recently been reviewed and both the ER and actin are linked to the recruitment and assembly of the constriction apparatus, which the authors term the “divisome” [46].

## 3. Mitochondrial Fusion

### 3.1. Demonstration of Mitochondrial Fusion

Fusion of isolated plant protoplasts allows the fusion of cells with different mitochondria and the production of cytoplasmic hybrid plants. Mitochondrial DNA recombination was demonstrated between two different mtDNAs in these latter studies [47]. Mitochondrial fusion with subsequent mitochondrial DNA recombination was recognised as a common phenomenon in somatic hybrid/cybrid plants [48]. It was much later that there was a direct demonstration of mitochondrial fusion in plants using the photoconvertible fluorescent protein Kaede targeted to the mitochondrion of onion epidermal cells [9]. Kaede targeted to the mitochondria causes a green fluorescence. A proportion of the mitochondria were photoconverted to red. In onion epidermal cells, green and red mitochondria fused transiently and became yellow and then the fused mitochondria divided [9]. Another study used the protoplast fusion approach. In this case, mitochondria from one protoplast fusion partner were labelled with the green fluorescent protein, while the other fusion partner contained red-staining MitoTracker-labelled mitochondria [18]. Fused mitochondria produced a yellow signal and showed what was called massive mitochondrial fusion (MMF), with the whole mitochondrial population undergoing fusion. It had been previously shown that isolated protoplasts destined for plant regeneration produce elongated mitochondria [7], which subsequently undergo fission prior to cell division (Figure 1). The protoplast fusion supported the elongated mitochondria being due to mitochondrial fusion. 

### 3.2. The Mechanism of Mitochondrial Fusion

Unlike the situation with fission, obvious orthologues of yeast or mammalian fusion have not been found [20]. However, recently, the GTPase ATMIRO2 has been investigated in tobacco epidermal cells [49]. Homologues in yeast (ScGEM1) affect mitochondrial–ER interactions and in mammals (HsMIRO1) affect mitochondrial motility. Evidence was obtained that AtMIRO2 regulates the tethering of mitochondria to the ER, such that ER–mitochondria attachment increases mitochondrial fusion, associated with increased clustering of mitochondria and decreased motility [49]. It has been shown that actin polymerisation is not required for mitochondrial fusion [18,50], though myosin and microtubule inhibitors reduced fusion [18]. White et al. [49] have also suggested a role for myosin in regulating mitochondrial fusion, based on analogies withHsMIRO1. Jaipargas and co-workers also found that the ER influenced mitochondrial fusion [5]. The important factors encouraging fusion were decreased tubular ER and mitochondrial motility and increased polygon size; in addition, myosin was suggested to be important. A mutant that affects mitochondrial clustering has been identified. This mutant known as *friendly* causes clustering of mitochondria because of extended association time between mitochondria [51]. Mitochondrial clustering is a prerequisite for mitochondrial fusion regulated by the *FRIENDLY* gene. Again, the importance of the regulation of mitochondrial motility comes to the fore [5,18,49,51]. The specific factors that enable the fusion of the mitochondrial membranes remain to be elucidated.

## 4. Significance of the Mitochondrial Fusion/Fission Cycle

### 4.1. Mitochondrial DNA Content per Mitochondrion Is Highly Variable

The fusion/fission cycle has meant that the mitochondria population in a cell should be thought of as a “discontinuous whole” [19]. What is the biological role of the mitochondrial fusion/fission cycle? The fusion/fission cycle has helped resolve one of the major historical problems of plant mitochondrial molecular genetics. It has been proposed for some time that plant mitochondria have variable amounts of DNA or no DNA at all [52] and this has subsequently been confirmed [8,17,53,54]. Fusion offers the opportunity for all mitochondria to gain access to mtDNA; tracking nucleoids provides evidence for this. Direct demonstration of fusion accompanied by nucleoid visualisation shows that mitochondrial fusion can decrease nucleoid heterogeneity, enabling most mitochondria to contain DNA [9,18]. Mitochondrial DNA is packaged into membrane-bound nucleoids, which are nucleoprotein structures readily visualised by the fluorochrome DAPI [12,18,55]. Even though it is possible to visualise nucleoids in most mitochondria after massive mitochondrial fusion [18], this does not necessarily mean all nucleoids contain a complete genome [17,54,56]. The mitochondrial genome can be very large [13] and is also multipartite, physically a mixture of linear, branched and fewer small circular forms [1,13,55,57]. However, the mtDNA maps to a large circular form using mapping and sequence assembly [13]. Fusion not only reduces mtDNA heterogeneity between mitochondria but allows mixing of the mitochondrial contents including mRNAs, proteins and metabolites. This mixing must be important as there are more mitochondria than there are copies of specific genes [8,58]. Different mitochondrial genes can have different copy numbers, consistent with the multipartite, subgenomic model [8,58], with not all subgenomic molecules being replicated to the same extent.

### 4.2. MtDNA Recombination 

It has become clear that, as originally proposed by Lonsdale et al. [52], the total mtDNA of the cell must be considered as a single entity. It is the capacity for mitochondrial fusion that allows the mtDNA population to participate in recombination—it cannot be facilitated in a single punctate mitochondrion. The recombination allows for rapid structural evolution but suppresses base sequence evolution [13,52,59,60]. Homologous recombination is driven by high numbers of repeated sequences. It is fascinating that plant mtDNA with its complex genome has lower base substitution rates than cpDNA or plant nuclear DNAs as well as animal mtDNAs [59,60]. It has been suggested that this is due to the genome facilitating homologous recombination-dependent repair and mismatch repair [13]. Therefore, despite the diversity of the mtDNA with its subgenomes, the genotype is faithfully transmitted from one generation to the next. Nevertheless, the mtDNA is transmitted as nucleoids [13]; however, they do not necessarily contain a whole genome [17,54,56]. Therefore, MMF is an important consideration which is developed further in the MMF section below.

### 4.3. Cytoplasmic Male Sterility

Mitochondrial DNA-encoded factors cause cytoplasmic male sterility (CMS), an important tool in the development of hybrid crops [61,62]. CMS commonly involves the transcription of open reading frames (*orfs*), which ultimately causes sterile pollen [61,62]. While this latter type of *orf* could derive from interspecific hybridisation and mtDNA rearrangements [62], following mitochondrial fusion, there are other types of CMS that can derive from interspecific somatic hybrids. One example is mitochondrial fusion and the development of feminised stamens (carpel-like); thus, there are no organs for pollen production [63]. The development of carpelloid stamens is associated with mtDNA recombination. The CMS phenomenon can clearly be linked to mitochondrial fusion, mtDNA recombination and mitochondria–nucleus compatibility. The inheritance of mitochondrial genotypes is generally maternally via the egg cell [64,65].

### 4.4. Mitochondrial Fusion and Energetics 

Jaipargas et al. [5] found that mitochondrial fusion was favoured under conditions of low energy status, such as darkness, low sugar and hypoxia, where increased energy levels are required. This raises the question of whether mitochondrial fusion or fission can be utilised to influence cellular metabolism. White et al. [49] suggest that fusion, promoted by ER tethering and low mitochondrial mobility, can be used as a device to support high energy demand. Possibly, increased mixing of mitochondrial contents optimises the capacity for ATP production. 

### 4.5. Mitochondrial Fusion and Evolution

A study by Rice et al. [66] has shown, quite dramatically, the role of mitochondrial fusion in horizontal gene transfer in the evolution of Angiosperms. The mtDNA (3.9 Mb) from the Angiosperm *Amborella trichopoda* mapped to five circular chromosomes, coming from Angiosperms, green algae and mosses. Following capture of the different genomes, there was recombination. It is argued that fungal or animal mtDNA does not feature due to the different mitochondrial fusion mechanisms that are common to Angiosperms, algae and mosses. The evidence obtained to support multiple mitochondrial fusion is based on very detailed sequence analysis.

## 5. Massive Mitochondrial Fusion in the Plant Life Cycle

### 5.1. The Metabolising Non-Dividing Cell 

In a metabolising cell not undergoing division or differentiation, there are reports of up to 600 punctate mitochondria in mesophyll cells [57], but this number can be much higher. In the onion epidermal cells used to study mitochondrial fusion by Arimura et al. [9], there were more than 10,000. The punctate mitochondria underwent fusion in a “kiss-and-run” type process. Typically, green and red labelled mitochondria fused transiently to form a yellow mitochondrion and separated to form two yellow mitochondria, with this process occurring throughout the cell until all the mitochondria in the cell were yellow in 1–2 h. This suggests a constant mixing of mitochondrial contents by fusion to ensure most mitochondria had the capacity for optimum ATP production. 

### 5.2. Mitochondrial Fusion and the Cell Cycle

Given that mitochondria are not derived de novo, they must grow and divide in the cell cycle as well as having an appropriate balance between fusion and fission. Mammalian mitochondria form a reticulate network by massive mitochondrial fusion in the cell cycle [67,68,69,70]. There is evidence in some cell lines that this occurs in G1 and the G1/S transition. This reticulum formation can be critical for normal cell cycle progression. Importantly, the reticulate mitochondria have a greater ATP production capacity than at any other stage [69]. There have been no reports of mitochondrial reticulum formation in the cell cycle of flowering plants. Wang et al. [45] have studied the distribution of mitochondrial size across the cell cycle in *N. tabacum* BY-2 cells. The percentage of the largest mitochondria is highest in interphase and the percentage of the smallest mitochondria is highest in anaphase. It was suggested that most fission occurs in mitosis. The mitochondrial fission timing is related to the phosphorylation and ubiquitination of the DRP3A/3B proteins. The DRP3A/3B proteins are most active in the phosphorylated state during mitosis and are partially degraded in interphase. This means that in interphase, there is more of a balance between fusion and fission, with the DRP3A/3B levels of expression contributing to the fusion/fission balance. In isolated *N. tabacum* protoplasts undergoing the first division, punctate mitochondria increased from 700 to about 2000 [7]. In protoplast division, mitochondria distribute throughout the cell via actin filaments, enabling actin-dependent partitioning of mitochondria in equal numbers [7] to daughter cells (Figure 1). In roots, just above the quiescent centre, mitochondria numbers double in the cell cycle and there is no MMF [54]. In this latter study, it was not investigated whether there were binary fusions as in onion epidermal cells [9], but presumably, there is similar fusion and fission of punctate mitochondria.

Mitochondrial DNA replication is required to maintain the genome. Available evidence is consistent with the RDR (recombination-dependent replication) replication mechanism involving linear molecules, similar to that used in bacteriophage T4 [20,55]. There are examples in Arabidopsis of mtDNA duplication (99 to 183 nucleoids), associated with an increase in mitochondrial number from 133 to 212, during the development of the two-cell embryo [71]. However, mtDNA replication may be highly amplified in some cells and then partitioned subsequently to daughter cells without mtDNA replication, as occurs in root growth [54,72]. When mitochondria undergo fission in the cell cycle, there is segregation of mtDNA to daughter mitochondria. Given the wide distribution of mtDNA levels in mitochondria, the fairly equal partitioning that occurs in chloroplasts [1] is unlikely to occur. Mitochondrial nucleoids are bound to the mitochondrial membrane [55,73]. Whether the nucleoids are distributed in the mitochondrion to assist equal partitioning is unclear. The cristae membrane structures may make this difficult. 

Given that mitochondria are not derived de novo, it is essential that the mitochondria transmitted to daughter cells in the cell cycle are of good quality. Fusion and fission contribute to this, but dysfunctional punctate mitochondria must be eliminated. There is now good evidence that plants [74,75], as with other eukaryotes have this capacity; though the mechanisms are similar, they are not identical [76]. Removal of dysfunctional mitochondria and chloroplasts can be carried out by autophagy [1,76,77], known as mitophagy for mitochondria. Mitochondria are sequestered by autophagosomes which are able to fuse with a vacuole, where the mitochondria are subsequently degraded. Dysfunctional mitochondria may be recognised by excessive ROS production (O_2_-), or possibly, a loss of membrane potential (Δ*ψ*_m_) [76,78]. Central to the autophagy process are the AUTOPHAGY-RELATED (ATG) proteins [76,78,79], which are involved in phagophore assembly, ultimately forming the double membrane autophagosome [78]. The phagophore membranes may originate from the ER, aided by several ATG proteins, notably the ATG1/ATG13 complex, the ATG5 complex and ATG8, which complexes to the phagophore membrane via phosphoethanolamine (ATG-PE form) [76,77]. What is implied here is that in the cell cycle is a type of surveillance mechanism for mitophagy. However, mitophagy and its regulation have been predominantly investigated in the context of a range of stressors, such as UV light [80], and senescence [76,77,78,79]. Interestingly, recent work has implicated the FRIENDLY protein, linked to mitochondrial fusion because of its clustering role, in mitophagy. It has been shown that *friendly* mutants are defective in mitophagosome formation, as FRIENDLY is not recruited to the damaged mitochondria [81].

### 5.3. Massive Mitochondrial Fusion in Regenerating Protoplasts

Isolated *N. tabacum* protoplasts, when isolated and cultured, can be readily regenerated into whole plants. Prior to the first division, the mitochondria undergo MMF [7] followed by fission (Figure 1). Essentially, the whole mitochondrial population changes from punctate mitochondria to predominantly long tubular structures, though there are examples of unusual tubular structures and large structures of variable shapes because of the fusion [7,18]. Subsequent fission results in numerous (approximately 2000) punctate mitochondria per cell. To check if this was indeed mitochondrial fusion, two isolated protoplasts were fused. In one protoplast, the mitochondria fluoresced green with GFP, while in the other, the mitochondria fluoresced red with MitoTracker [18]. After protoplast fusion, mitochondrial fusion resulted in large yellow structures as well as tubular structures.

Studies were carried out with the protoplast system to investigate mitochondrial nucleoid heterogeneity pre- and post-fusion. In freshly isolated *N. tabacum* protoplasts, prior to MMF, approximately 25% of the mitochondria did not stain with DAPI, with no nucleoids visible. When the same analysis was performed post-fusion, almost all mitochondria contained nucleoids visualised by DAPI. This study showing that mitochondrial fusion decreased nucleoid heterogeneity is consistent with the mitochondria fusion studies by Arimura et al. in onion epidermal cells [9].

MMF was also demonstrated in cultured *Medicago truncatula* and *Arabidopsis* mesophyll protoplasts but not protoplasts from *N. tabacum* callus and BY-2 cells [18]. In the case of the *N. tabacum, M. truncatula and Arabidopsis* mesophyll cells, the MMF was associated with dedifferentiation and reprogramming, while the callus and BY-2 cells were already dedifferentiated. It is plausible that MMF in cells starting a new asexual generation reflects a need to maximise mitochondrial and DNA quality. Yet, what about MMF in the plant sexual life cycle?

### 5.4. Massive Mitochondrial Fusion in the Shoot Apical Meristem

Seguı-Simarro et al. [10] examined mitochondria morphology in collected EM images from the Staehelin laboratory from many *Arabidopsis* cell types. The EM images were largely from root tip, stem, mature leaf, meiocyte, microspore, pollen, endosperm and embryo. All the cells had mitochondria with the classic punctate type of morphology. However, cells of the shoot apical meristem (SAM) and the leaf primordia possessed a large perinuclear mass of fused mitochondria. Particularly at the latter stages of the cell cycle, in G2 and early mitosis, they possess a large tentacular/cage-like mitochondrial structure surrounding the nucleus and then the spindle. The large tentacular structure forms at the G2/mitosis stage because of both fusion and growth, so mitochondrial mass doubles in the cell cycle. After cytokinesis, each daughter cell receives a tentacular mitochondrion. Subsequently, mitochondrial fission produces punctate mitochondria. It was concluded that the mitochondrial changes in these SAM cells ensure high rates of mtDNA recombination and equal partitioning of mitochondria to daughter cells [10].

Subsequently, Seguı-Simarro and Staehelin argued [82] that what was happening in the SAM, and not root meristems, for example, was extensive mixing of the mtDNA and mitochondrial contents. This ultimately leads to high-quality mitochondria and mtDNA in the female gametes. In the development of the vegetative parts of the plant from the SAM, cell lineages derived from the SAM have normal punctate mitochondria. The cell cycle in the non-SAM cells do not have a mtDNA reticulate phase as in mammalian cells. The SAM studies [10,82] suggest that this MMF that occurs in the SAM contributes to a form of quality control of mitochondria for the next generation, as observed in regenerating protoplasts. 

### 5.5. Massive Mitochondrial Fusion in the Zygote

Mitochondria in the egg cell and zygote have recently been examined in *Arabidopsis*. The egg cell has a mix of punctate and tubular mitochondria. However, the zygote has extremely long tubular mitochondria, most likely due to MMF [83]. These long tubular mitochondria form in association with long F-actin filaments. This again emphasises the importance of the cytoskeleton in the fusion process. Just prior to the first asymmetric cell division, the mitochondria fragment into small punctate mitochondria. The apical cell inherits small, largely punctate mitochondria, while the basal cell which forms the suspensor has the tubular mitochondria. This suggests that the punctate mitochondria are associated with development of the embryo. In animal cells, exit from the cell cycle and entry into differentiation may be associated with inhibition of fusion and the formation of fragmented mitochondria [84]. There are specific examples of this. In neurogenesis, mitochondrial fusion is associated with cell renewal, whereas cells that differentiate into neurons have high levels of mitochondrial fission [85].

### 5.6. Massive Mitochondrial Fusion in Germination

Mitochondria have been studied by Paszkiewicz et al. [86] in the dry seed, through imbibition and during germination in *Arabidopsis*. In the dry seed, the mitochondria are rudimentary, with little internal membrane development and are called promitochondria. Nucleoids are present in 90% of the promitochondria. It is feasible that the MMF that occurs in the SAM [10,82] and in the zygote [83] contributes to the nucleoid content being less heterogeneous in the promitochondrial population. During germination, there is MMF in the form of tubuloreticular mitochondria that surround the nucleus, similar to what occurs in the SAM. This structure is associated with a doubling of mitochondrial volume. Subsequently, the tubuloreticular structure undergoes fission and the number of mitochondria is double the number in the dry seed. In this case, the number of mitochondria without nucleoids decreases with nucleoids being observed in 67% of the mitochondria. This re-establishes the heterogeneous nucleoid situation, where part of the population of mitochondria lacks nucleoids [8,17,53,54] and “kiss and run” fusion becomes important [9]. The germination study again points to the importance of MMF in the potential for recombination, DNA repair and mitochondrial content mixing, as autotrophy, growth and development are initiated. 

### 5.7. Significance of Massive Mitochondrial Fusion

Given that the total mtDNA of the cell must be considered as a single entity, this makes MMF or hyperfusion an important part of maintaining the integrity of the mitochondrial genome. This means that it provides an important opportunity for all the subgenomes to interact for recombination and DNA repair for the next generation. What is known currently is that MMF occurs in the SAM [10,82] where flowering is initiated, in the zygote [83] and in germination [86], which are key points in the life cycle. This is not to say that the fusion/fission cycle involving few mitochondria is not unimportant in the cell cycle, cell development and the functioning of the cell. In these latter cases, the importance may be in DNA replication, and ensuring transcripts, proteins and metabolites are readily available for the maintenance of functional mitochondria and their genomes.

While the fusion/fission cycle is of key importance for maintaining mitochondria and their genome, there may be other roles for MMF. In plants, there is some evidence that fusion favours high energy demand [5,49] and MMF occurs at times prior to the onset of major development shifts. MMF also occurs prior to the first cell division on the path to regeneration. If there is a connection between MMF and ATP production, there may be a role for manipulating mitochondrial fusion as an approach to modulating mitochondrial performance [49]. In mammalian cells, there is evidence that the hyperfused mitochondrial reticulum in the G1/S stage of the cell cycle produces more ATP than any other stage of the cell cycle [69].

## 6. Conclusions

**Figure 2:**
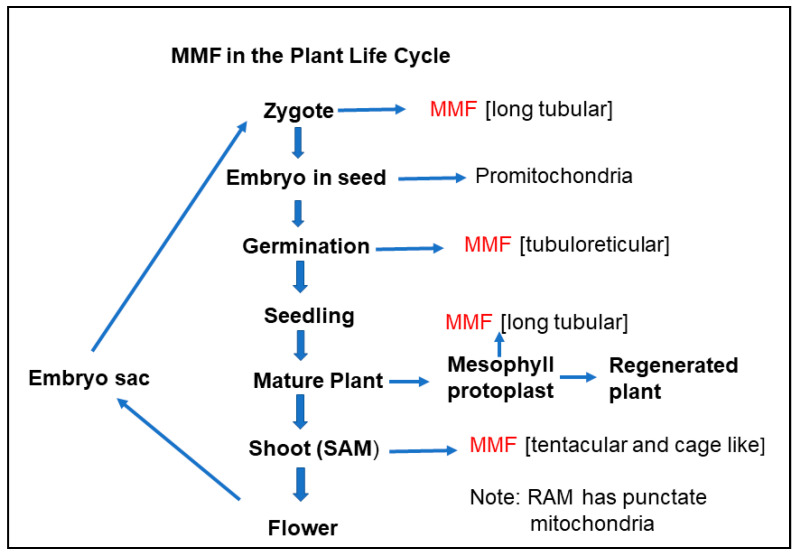
Massive mitochondrial fusion (MMF) in the plant life cycle. Diagram based on information presented in Section 5, mainly from references [7,18,82,83,86].

The fusion and fission of plant mitochondria are crucial to maintaining the integrity and quality of the mitochondrial genome. This is because the relatively large mitochondrial genome must be considered on a whole-cell basis. In most species, plant mitochondria have a heterogeneous DNA content, and a single mitochondrion does not contain a whole genome. The fusion process allows critical mixing to facilitate recombination repair and reduce nucleotide changes in the critical genes required for ATP production. MMF or hyperfusion occurs at critical times of the life cycle, prior to flowering, in the enlarging zygote and at germination (Figure 2). MMF is also important in plant regeneration (Figure 2). The enhanced mixing of mitochondrial contents may also influence ATP production. It is possible that the MMF state boosts energy production prior to the initiation of major developmental change. While there is a reasonable understanding of critical genes involved in fission (such as DRP3A and 3B), understanding has lagged in the fusion process, though there are promising recent developments [49]. Exactly how the ER and cytoskeleton contribute to plant mitochondrial fission and fusion remains an interesting cell biology question.
